# Trust and emotional difficulties in adolescence: findings from a Swedish cohort study

**DOI:** 10.1093/eurpub/ckac129.524

**Published:** 2022-10-25

**Authors:** S Brolin Låftman, V Östberg, J Raninen

**Affiliations:** 1 Department of Public Health Sciences, Stockholm University, Stockholm, Sweden; 2 Department of Clinical Neuroscience, Karolinska Institutet, Stockholm, Sweden; 3 Centre for Alcohol Policy Research, La Trobe University, Melbourne, Australia

## Abstract

**Background:**

Trust is a key component of a socially sustainable society, and is typically divided into general trust (referring to trust in other people) and institutional trust (referring to trust in the public institutions of society). Trust tends to be developed and formed early in life. While a plethora of research has reported positive links between trust and health in adults, the aim of this study was to examine the associations between general and institutional trust and emotional difficulties in mid and late adolescence.

**Methods:**

Data were derived from the Swedish cohort study Futura01, using information collected amongst 3622 grade 9 students (∼15-16 years, t1) who were followed-up two years later (∼17-18 years, t2). General and institutional trust was measured by indices based on five items each at both t1 and at t2 (range 1-4). Emotional difficulties were measured by the Strengths and Difficulties Questionnaire (SDQ) subscale at both t1 and at t2 (range 0-10). Control variables included family type and cash margin at t1 and upper secondary school program (academic vs. vocational) at t2. Linear regressions were performed using the first difference (FD) approach, analysing the change in emotional difficulties (t2-t1) regressed on the change in general and in institutional trust (t2-t1), respectively.

**Results:**

Analyses simultaneously adjusting for change in both dimensions of trust showed inverse associations between the change in general trust and the change in emotional difficulties (b=-0.21, 95% CI -0.39, -0.04) and between the change in institutional trust and the change in emotional difficulties (b=-0.22, 95% CI -0.35, -0.09).

**Conclusions:**

Increases in general and in institutional trust between ages 15-16 and 17-18 years were associated with a corresponding decrease in emotional difficulties. The findings suggest that trust is a social determinant of emotional difficulties in adolescents. Endeavours to enhance trust in this age group are relevant.

**Key messages:**

• Changes in trust were inversely associated with changes in emotional difficulties in adolescents.

• The findings indicate that efforts to reinforce trust in adolescents are relevant.

